# Evaluation of a nurse mentoring intervention to family caregivers in the management of delirium after cardiac surgery (MENTOR_D): a study protocol for a randomized controlled pilot trial

**DOI:** 10.1186/1745-6215-15-306

**Published:** 2014-07-30

**Authors:** Tanya Mailhot, Sylvie Cossette, Anne Bourbonnais, José Côté, André Denault, Marie-Claude Côté, Yoan Lamarche, Marie-Claude Guertin

**Affiliations:** Faculty of Nursing, University of Montreal, C.P. 6128 succ. Centre-ville, Montreal, Quebec H3C 3J7 Canada; Montreal Heart Institute Research Center S-2490, 5000 Belanger Street, Montreal, Quebec H1T 1C8 Canada; Centre de recherche de l’Institut Universitaire de Gériatrie de Montréal, 4545 chemin Queen-Mary, Montréal, Quebec H3W 1W4 Canada; Centre Gératrique Maimonides Donald Berman, 5795 Caldwell ave, Montreal, Quebec H4W 1W3 Canada; Centre de Recherche du Centre Hospitalier de l’Université de Montréal, 900 Saint-Denis Street, Montreal, Quebec H2X 0A9 Canada; Department of Anesthesiology, Montreal Heart Institute, 5000 Belanger Street, Montreal, Quebec H1T 1C8 Canada; Department of Psychosomatics, Montreal Heart Institute, 5000 Belanger Street, Montreal, Quebec H1T 1C8 Canada; Department of Cardiac Surgery, Montreal Heart Institute, 5000 Belanger Street, Montreal, Quebec H1T 1C8 Canada; Montreal Health Innovations Coordinating Center, 4100 Molson street suite 400, Montréal, Quebec H1Y 3N1 Canada

**Keywords:** Bandura, Delirium management, Family caregivers, Nursing interventions, Pilot study, Randomized, Self-efficacy, Watson

## Abstract

**Background:**

Despite the use of evidence-based preventive measures, delirium affects about 40% of patients following cardiac surgery with the potential for serious clinical complications and anxiety for caregivers. There is some evidence that family involvement as a core component of delirium management may be beneficial since familiarity helps patients stay in contact with reality, however, this merits further investigation. There is also currently a gap in the scientific literature regarding objective indicators that could enhance early detection and monitoring of delirium. Therefore, this randomized pilot trial examines the acceptability, feasibility, and preliminary efficacy of an experimental nursing intervention to help family caregivers manage post-cardiac surgery delirium in their relatives. It also explores the validity of a new and innovative measure that has potential as an indicator for delirium.

**Methods/Design:**

In this two-group randomized pilot study (n = 30), the control group will receive usual care and the intervention group will receive the experimental intervention aimed at reducing delirium severity. The intervention nurse’s objective will be to foster the family caregiver’s self-efficacy in behaving in a supportive manner during delirium episodes. Data will be collected from standard delirium assessment scales and a novel measure of delirium, i.e., cerebral oximetry obtained using near infrared spectroscopy, as well as medical records and participants’ responses to questionnaires.

**Discussion:**

New strategies for early detection, monitoring, and management of delirium are needed in order to improve outcomes for both patients and families. The present article exposes feasibility issues based on the first few months of the empirical phase of the study that may be useful to the scientific community interested in improving the care of patients with delirium. Another potentially important contribution is in the exploration of cerebral oximetry, a promising measure as an objective indicator for early detection and continuous monitoring of delirium. The proposed pilot study will build towards a larger trial with the potential to improve knowledge about delirium management and monitoring.

**Trial registration:**

This pilot study was registered at Controlled Trials on March 27th 2013 and was assigned #ISRCTN95736036.

**Electronic supplementary material:**

The online version of this article (doi:10.1186/1745-6215-15-306) contains supplementary material, which is available to authorized users.

## Background

A large number of patients require cardiac surgery each year and up to 40% of them will experience postoperative delirium [1]. Delirium is a neuropsychological syndrome characterized by fluctuations between acute agitation (hyperactivity) or lethargy (hypoactivity) [2]. According to the Diagnostic and Statistical Manual of Mental Disorders, Fifth Edition (DSM-V), patients may be confused and disoriented [2]. Incoherent speech, visual or auditory hallucinations, and labile emotions may also be observed. Delirium usually results from a mix of predisposing and precipitating factors (e.g., older age, tobacco and/or alcohol consumption, and cognitive decline, as well as surgery, pain, dehydration, and social isolation) [3–5]. Some of these factors can be addressed with preventive clinical interventions, but others cannot and require different approaches to manage and monitor the delirium episode.

Hyperactive delirium requires urgent care because patients may pull out endotracheal tubes or chest drains, injure themselves by falling, and sometimes even sustain fractures. On the opposite extreme, hypoactive delirium may interfere with essential care activities, such as daily breathing exercises, to prevent respiratory infections, or walking exercises to prevent loss of functional abilities. Such clinical complications of delirium contribute to longer length of hospital stay, increased mortality risk, and long-term psycho-functional sequelae [1, 2, 5–7]. Family caregivers of patients with delirium are also greatly affected. Witnessing these behavioral and emotional manifestations of delirium in a loved one has been described by caregivers as highly disturbing, with 75% of families reporting anxiety [8–10].

While clinical guidelines on delirium prevention, management, and monitoring have been developed based on meta-analyses, systematic reviews, and clinical studies [2, 11, 12], the level of evidence is much stronger for prevention rather than other interventions. Authors undertaking meta-analyses on delirium prevention have concluded that strategies such as interdisciplinary work and health care staff training focusing on geriatrics and delirium has led to decrease in the delirium incidence (odds ratio: 0.64; 95% confidence interval (CI): 0.46–0.88) [13]. Although less research has focused on the management strategies for delirium, certain strategies have shown efficacy in decreasing mortality, duration of delirium, and length of hospital stay, as well as speeding up post-delirium psycho-functional recovery in older patients hospitalized in medical wards [14, 15]. These later strategies include a combination of pharmacological (e.g., antipsychotics) and non-pharmacological (e.g., reality orientation, reassurance) interventions.

Despite the lack of strong studies in delirium management, guidelines suggest focusing on two main objectives: assessment for probable causes and simultaneous management of delirium manifestations [2]. However, certain settings, such as intensive care units (ICUs), make it more challenging to manage delirium optimally with a non-pharmacological approach. Indeed, ICU patients are in very precarious states, often falling in and out of consciousness, and requiring highly technological and invasive care that needs to be prioritized to insure survival. In this context, it is challenging for nurses to acquire detailed knowledge of patients’ personal backgrounds and personalities, thus limiting the personalization of delirium management interventions with reality orientation and reassurance.

The presence of family members is also central in delirium management guidelines, since familiarity helps patients stay in contact with reality – but the intensive care setting may limit this important aspect of care. Family caregivers’ involvement is a gold standard in other contexts of care such as community health, pediatrics, geriatrics, and palliative care [16]. For example, in patients with dementia (a condition with behavioral manifestations somewhat similar to those accompanying delirium), family caregiver involvement in dementia management has been associated with reduced severity of agitation and aggressive behaviors [17]. However, family caregiver involvement in delirium has been sparsely studied. Black et al. [18] involved family caregivers in “psychological care” in a comparative time series study with 170 adult acute care patients (87 with facilitated caregiver participation and 83 without facilitated caregiver participation), 77 of which developed delirium. It was hypothesized that having caregivers talk with patients, offer reality orientation, or hold their hand and reassure them at the bedside would potentially prevent delirium and enhance psycho-functional recovery. Results showed no significant difference in delirium prevention with both groups showing similar occurrence, although a superior psycho-functional recovery was observed in patients with facilitated caregiver participation in comparison of those without [18]. In two other studies [12, 14], caregivers were encouraged at the bedside of patients with delirium [14] and were involved in discussing the discharge plan with the health care team [12]. However, these two studies did not report the results specifically related to caregiver involvement and were not conducted with cardiac surgery patients.

These studies open the door to a novel way of delivering more personalized non-pharmacological delirium management interventions in ICU settings. The involvement of a caregiver who knows about the patients’ personality, life, and family, may offer a reassuring presence, thereby contributing to reality orientation. The present paper describes a study protocol of a delirium management nursing intervention involving caregivers of post-cardiac surgery patients with the hope of diminishing delirium severity and its related clinical complications as well as improving caregiver psychological outcomes. The protocol also tests the validity of regional oximetry (_r_SO_2_) measures obtained with near infrared spectroscopy (NIRS) which may provide an objective indicator for early detection and continuous monitoring of delirium. Finally, several feasibility issues such as delirium detection, risk factors for delirium, and ethics challenges with informed consent are discussed.

### Study objectives and research hypotheses

The primary objective of this pilot study is to examine the acceptability and feasibility of i) the study design; ii) the experimental nursing intervention; and iii) a novel measure, cerebral _r_SO_2_ obtained by NIRS, a non-invasive method to detect cerebral oxygen imbalances among patients with delirium as well as its validity among patients with delirium.

The secondary objective of this pilot study is to examine the preliminary effect of the intervention on patient and caregiver outcomes. The following are the research hypotheses for the study’s secondary objective:

For patient outcomes

Compared with controls, patients in the intervention group will present:

1. Less severe delirium in the 3 days following onset;
2. Fewer complications (defined as either a sternal wound dehiscence, fall, respiratory tract infection, or accidental removal of urinary catheter, drain, arterial line or endotracheal tube) in the 3 days following onset of delirium;
3. Shorter total length of ICU and total hospital stay;
4. Enhanced psycho-functional recovery one month after the onset of delirium;

For caregiver outcomes

In comparison with controls, caregivers in the intervention group will present:

(H5) A lower anxiety level after the intervention (Day 4 following delirium onset), at Day 15, and at 1 month following delirium onset;
(H6) A higher sense of self-efficacy after the intervention (Day 4 following delirium onset), at Day 15, and at 1 month following delirium onset.

### Trial design

A randomized pilot study is proposed to test this novel delirium management intervention delivered in collaboration with family caregivers. This pilot study is registered at Controlled Trials (#ISRCTN95736036) and was approved (Reference number: 2012–288, 1420) by the Scientific and Ethics Committee of the Montreal Heart Institute Research Center.

## Methods

### Setting

An academic hospital in Canada was selected as the research hospital. In this milieu, cardiac surgery patients are generally hospitalized in the ICU for 1 to 2 days following surgery and then transferred to the post-ICU surgery ward for the following 3 to 4 days before being discharged from hospital.

### Sample: eligibility criteria

Patients and family caregivers must be 18 years or older, have the ability to speak and read French, and have the physical and cognitive ability to give informed consent. Additionally, all patients must be scheduled for coronary artery bypass grafting (CABG) or heart valve surgery, must spend their full postoperative stay in the study hospital (i.e., not be transferred to another hospital), and present postoperative delirium defined by a score of 4 or higher on the Intensive Care Delirium Screening Checklist (ICDSC) [19] and confirmed by a medical diagnosis.

Eligible caregivers will be identified as such by the patient, and must be available twice daily for 3 consecutive days after delirium onset to visit the patient and receive pre- and post-bedside visit interventions.

### Intervention

#### Control group

Usual care provided to the control group includes delirium prevention and management interventions such as early mobilization and post-surgical pain control. Generally, patients with delirium are referred to psychiatric services for management of the appropriate medication regimen throughout the delirium episode. In addition, the research hospital regularly offers the staff a 3-hour training session in delirium management, which includes recognition of predisposing and precipitating factors of delirium, the ICDSC screening tool [19], and an update on nursing interventions from clinical practice guidelines on delirium. At the onset of delirium, caregivers receive a document with a brief explanation of delirium and its causes. After surgery, families may visit patients in the ICU for 15 minutes per hour, and in the post -ICU ward anytime between 9 am and 9 pm.

#### Intervention group

The proposed experimental intervention consists of mentoring family caregivers about delirium management behaviors and offering them the necessary support to adequately adopt these behaviors while at the bedside of a loved one with delirium. The experimental intervention was designed to ensure that intervention components and structure would be relevant both when delivered on the ICU ward or on the post-ICU surgery ward. For instance, family visit rules are different in each setting and the intervention design was planned to be feasible in both settings.

#### Intervention structure

The intervention structure is described in Table 1 and was based on previous studies with caregiver involvement with delirium [18, 20] or dementia [17] and on the level of involvement that was deemed acceptable to family caregivers and acute care patients who are in a precarious state after cardiac surgery. The duration of each encounter was selected to allow time for the caregiver to become comfortable with behaviors within the timeframe allowed for visiting hours and to allow time to reflect on the bedside visits. The intervention consists of a total of seven encounters involving the nurse, caregiver and patient. The first encounter will begin within 24 hours of delirium onset followed by five others at a rate of two encounters a day over the next three consecutive days. A seventh encounter will take place just before hospital discharge. The duration of each of the first six encounters is planned as follows: a pre-bedside mentoring session of 30 minutes; a 15 minute bedside visit with the patient (to abide by the ICU’s visitation hours policies); and a post-bedside session of 15 minutes. The seventh encounter before discharge will last 30 minutes. The nurse-mentor accompanying the caregiver will act as his mentor and is a nursing PhD candidate experienced both with cardiac surgery patients and delirium. In addition, she has received the same 3 hours of training offered by the research hospital to the staff on a regular basis.

**Table 1:** **Schedule of enrolment, interventions and assessments**

Study timepoints	Study period: ***A template is adapted from the SPIRIT guidelines***[21]								
	Enrolment	Allocation		Post-allocation					Close-out
	***-t***_***1***_	0	***t***_***1***_	***t***_***2***_	***t***_***3***_	***t***_***4***_	***t***_***5***_	***t***_***6***_	***t***_***7***_
Participant timeline	***Delirium onset***		***Day 1***	***Day 2***	***Day 3***	***Day 4***	***Discharge from hospital***	***Day 15***	***Day 30***
*Enrolment:*									
Eligibility screen/Informed consent:									
Caregivers	√								
Patients							√		
Allocation of participants		√							
*Intervention encounters:*									
Control group									
Intervention group			√	√	√		√		
*Assessments:*									
Baseline using a sociodemographic questionnaire	√								
1. Primary Objectives: Acceptability and feasibility of:									
1.1 –the study design	*	*	*	*	*	*	*	*	*
1.2 –the intervention			*	*	*	*	*		
1.3 –rSO2 using the INVOS 5100 device from Covidien, Mansfield MA USA			√	√	√				
2. Secondary Objectives									
H1- Delirium Severity assessed using the Delirium Index scale [22]			√	√	√				
H2- Complications obtained from a keyword search in the medical chart									√
H3- Length of stay obtained from the medical chart									√
H4- Recovery assessed using the Sickness Impact Profile scale [23, 24]									√
H5- Anxiety assessed with the State Trait Anxiety Inventory State [25]	√					√		√	√
H6- Self-efficacy assessed with a scale adapted from Bandura [26]	√					√		√	√

√: Research activity or questionnaire completed at this timepoint.

*: Assessed with indicators detailed in Table 4.

#### Intervention content

We used Human Caring Theory [27, 28] to guide the overall nurse-caregiver-patient interactions. This theory promotes transpersonal caring relationships between nurses, patients, and families. In order to build such relationships, the nurse is required to be respectful and open to both learning from patients and caregivers as well as investing the necessary effort to understand patients and caregivers’ perceptions and realities [27]. The Human Caring Theory will form the context in which caregivers will learn how to be a reassuring presence at the patient’s bedside, thus enabling their effective involvement in delirium management.

The intervention also relies on Bandura’s Social Cognitive Theory [29] because a caregiver’s involvement in delirium management depends on self-confidence in adopting specific behaviors. This theory suggests four basic elements on which individuals rely to assess their perception of self-efficacy in adopting and maintaining new behaviors: i) other people’s performances (vicarious experience, role modelling); ii) other people’s feedback (verbal persuasion); iii) personal experiences (performance accomplishment); and iv) their emotional state.

Finally, we integrated a bidirectional learning context in the present intervention because it was proposed that both the nurse and the caregiver will combine their respective knowledge and experience to optimize the delirium management strategies. This learning context implies an active partnership from the caregiver and nurse which corresponds to Anderson’s description of mentoring [30]. The nurse mostly refers to scientific knowledge on delirium and its management while the caregiver mostly refers to personal knowledge of the patient as well as previous experience with delirium. Pre-bedside encounters between the nurse and caregiver will first serve to share each other’s knowledge of delirium and of the patient as well as to identify possible behaviors that may be effective to manage the actual delirium manifestation. In these same encounters, a goal of behavior adoption will be set and a plan will be made for a 15 minute bedside visit. As shown in Table 2, elements of each component of the theoretical framework will be part of the different encounters between the nurse and caregiver. In our experimental intervention, the nurse-mentor will facilitate an active partnership from the caregivers in their learning of new delirium management behaviors by involving them in the planning of bedside visits and by encouraging them to experience the learned behaviors [31]. The seven encounters will give the nurse-mentor and caregiver multiple opportunities for mentoring and applying the learned delirium management behaviors.

**Table 2:** **Experimental intervention content**

Time of encounter	Summary of content
Pre-bedside visit	• Share knowledge on delirium
	• Share information on present delirium situation (what has the caregiver observed in his/her previous visit and what has the bedside nurse shared with the nurse-mentor)
	• Discuss possible appropriate behaviors to retain (from Table 3)
	• Set a common goal on which behaviors will be adopted by the caregiver (ensure that the behavior agreed on with the caregivers corresponds to his/her abilities)
	• Share examples of the chosen behaviors
Bedside visit	• Nurse adopts discussed behaviors with the patients for the caregiver to observe (role model – vicarious experience of the caregiver)
	• Nurse encourages the caregiver to behave in a similar manner (performance accomplishment)
	• Provide reinforcement and positive feedback (verbal persuasion)
Post-bedside visit	• Nurse provides feedback on behavior
	• Reflect on the bedside visit, on the positive or negative impact of the behavior
	• Prepare for the next visit
	• Sixth encounter: an action plan for the caregiver will be planned with the nurse for the remainder of the patient’s hospital stay
Pre-discharge	• Seventh encounter: nurse, caregiver and patient discuss their experience with delirium

As presented in Table 3, a range of behaviors will be discussed with the caregivers. They will be informed that delirium-related manifestations can fluctuate from one moment to another, that they should plan a range of delirium management behaviors, and that they should be open to the possibility of changing and/or adapting the planned behavior(s) depending on the patient’s condition. A booklet containing examples of behaviors adapted for various delirium situations will be provided by the nurse-mentor, who will facilitate the learning of these behaviors. The caregivers will be encouraged to add any ideas of possible behaviors (e.g., family pictures, sharing a significant personal experience) they may consider appropriate to discuss with the nurse. The intervention content will be adapted according to the evolution of the delirium episode, for instance, if the delirium is resolved before the sixth encounter, the nurse will focus on facilitating general supportive behaviors instead of specific delirium management strategies.

**Table 3:** **Behaviors proposed at the bedside**

Categories	Examples of behaviors*
Being attentive	• Observe signs of pain (grimacing, avoiding movements, breathing short, and fast)
	• Report these observations to the nurse responsible for the patient
	• Check the proper use of hearing or visual aid if applicable
	• Be attentive to the signs associated with delirium (e.g., a person who does not know where she is, who sleeps a lot)
	• Observe signs of delirium such as agitation (a person who tries to pull out tubing)
	• Adopt a calm attitude in case of agitation
Maintain contact with the patient	• Speak in short, simple sentences
	• Use closed questions
	• Repeat information as necessary
	• Reduce distractions
	• Avoid confrontation
	• Validate patient’s expressed emotions
Be a reassuring presence and support	• Provide stimulating activities when appropriate (sit down for meals, breathing exercises)
	• Be present or call every day
	• After delirium: talk about the experience with the patient

*The proposed behaviors are based on several studies [4, 5, 16, 32–38] and clinical guidelines [7, 39–41].

In summary, the intervention content relies on a caring-learning relationship between the nurse and caregiver where both participants are actively involved in learning strategies designed to enhance the caregiver’s perception of self-efficacy and decrease his anxiety in adopting delirium management behaviors.

### Outcomes

Variables described in Table 4 will be used to evaluate the acceptability and feasibility of the study design and intervention.

**Table 4:** **Assessment variables for acceptability and feasibility**

Variables	Acceptability	Feasibility
	Indicators	
**Relative to the study design**		
(1) Caregiver and patient recruitment	• Primary indicator: obtaining consent from at least 75% of the caregivers approached to participate in the study	• Recruitment time
	• Percentage of eligible people who were included in the study	• Effectiveness of recruitment strategies
	• Caregiver reasons for refusal to participate in the study	• Number of potential participants
	• Difficulties in obtaining patient consent	
(2) Randomization	• Reasons for patient refusal to participate after the delirium episode	• Number of non-eligible participants randomized
(3) Participant retention	• Reasons for withdrawal from the study	
(4) Data collection	• Rate of completion of questionnaires	• Percentage of outcome measures collected
	• Reasons for non-completion of questionnaires	• Time required for completion of outcome measure tools
		• Respect of the data collection plan
		• Validity of cerebral oximetry as an indicator of delirium
(5) Between-group contamination	• Rate of participants in the control group exposed to the intervention (overlapping time for the intervention and usual care, e.g., because of prolonged hospital stay)	
		• Attendance rate of caregivers at the bedside
		• Moments of the day for which caregivers were present
		• Number of family members, other than the caregiver, who visited the patient
		• Relationships between visitors and the patient
		• Total time spent at the bedside by the caregiver
		• Total time spent at the bedside by other visitors
***Relative to the intervention***		
(1) Satisfaction of primary caregivers with the intervention	• The scores on the Treatment Acceptability and Preference Questionnaire (TAP) [42], completed on day 4 after delirium onset will help assess caregivers appreciation of the intervention. This questionnaire contains 5 items on which appreciation is scored using Likert scales, the higher the scores the more appreciation. The reported α is ≥0.80.	
	**Intervention group only**	
(2) Availability necessary to provide the intervention		• Total amount of time allocated to each contacts provided through the intervention
		• Duration of the presence of the intervention
		• Duration of the presence of the primary caregivers for the intervention
		• Moments of the day which took place at the bedside visits on T1, T2, and T3
		**Intervention group only**
(3) Material resources		• Costs relative to printed documents and electrodes used to measure cerebral oximetry
(4) Fidelity to the components and structure of the intervention	• Number of encounters received/planned	• Modalities of intervention planned versus received
	• Reasons for encounters not received	
	I**ntervention group only**	**Intervention group only**
(5) Selection and use of delirium management behaviors	• Number of suggested behaviors in comparison to adopted behaviors by the primary caregivers	• Clinical condition of the patient at each visit
	**Intervention group only**	• Number and type of behavior adopted by the intervention provider at each visit on T1, T2, and T3
		• Number and type of behavior adopted by the primary caregiver at each visit on T1, T2, and T3
		**Intervention group only**

The primary objective of this pilot study is to examine the acceptability and feasibility of the study design, the experimental nursing intervention and a novel measure.

To evaluate the acceptability and feasibility of the study design, the following variables will be measured throughout the study: i) caregiver and patient recruitment; ii) randomization; iii) participant retention; iv) data collection; and v) between-group contamination. These were selected based on randomized pilot study evaluation criteria developed by Sidani and Braden [43], as well as other evaluation criteria. The primary indicator that will help assess the acceptability of the study design relates to the variable relative to caregiver and patient recruitment. This primary indicator is to obtain consent from at least 75% of the caregivers approached to participate in the study.

The acceptability and feasibility of the nursing intervention will be evaluated using measures inspired by the work of Sidani and Braden [43] including: i) satisfaction of primary caregivers with the intervention; ii) availability to provide the intervention; iii) material resources; iv) fidelity to the content and structure of the intervention; and v) selection and use of delirium management behaviors. Description of the nursing intervention will be done by using a log of visits by the caregivers and other family members and of the behaviors they adopted, with any comments describing the patient’s reaction. All nurse-caregiver encounters will be described using a pre-coded possible behaviors list with additional space for incoming new behaviors.

**Figure 1:**
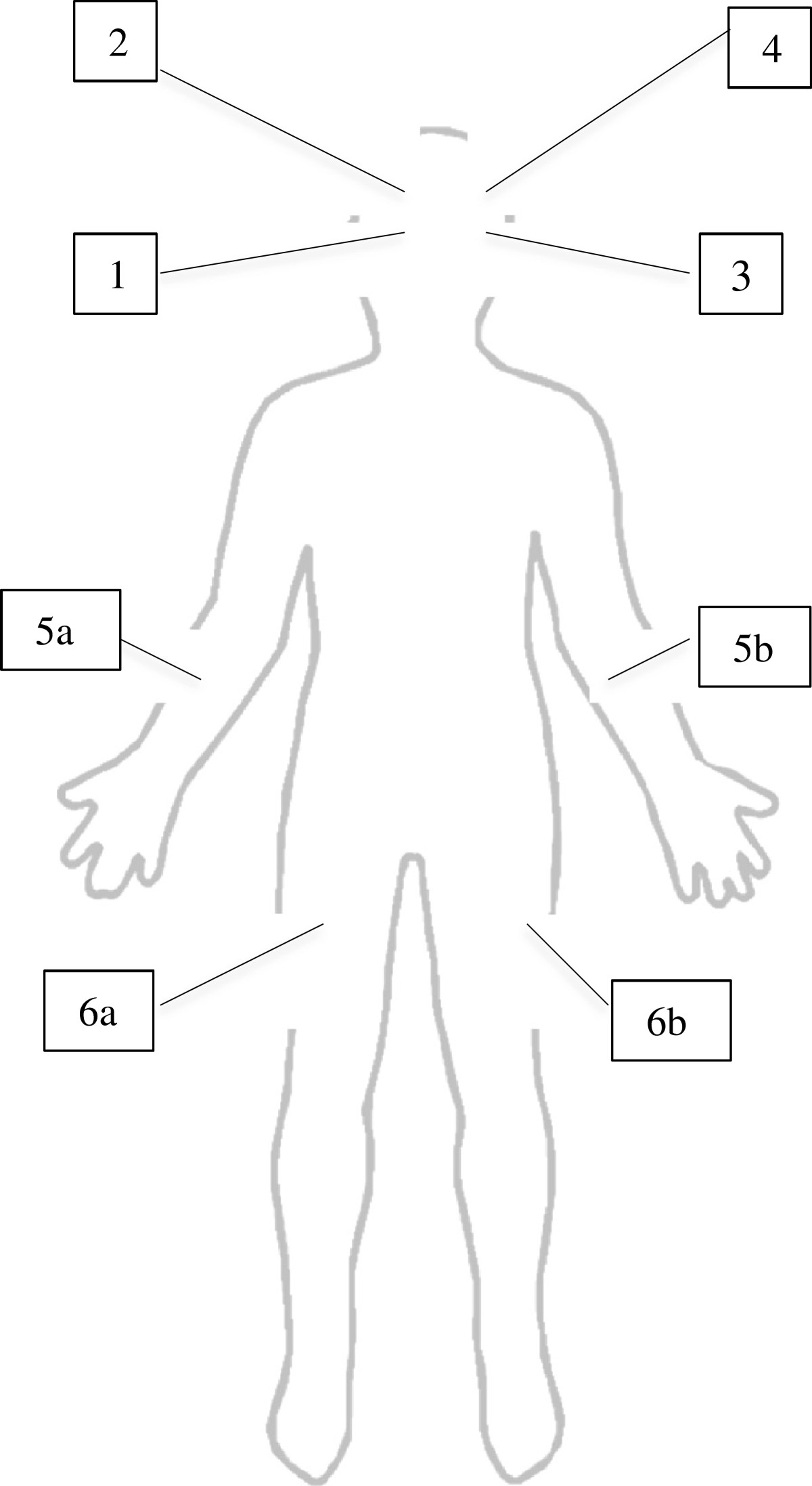
**Oximetry measures sites.** In this figure are represented the six different measuring sites from which oximetry values were collected. Lower right frontal cerebral (1), upper right frontal cerebral (2), lower left frontal cerebral (3), upper left frontal cerebral (4), and the possible areas for oximetry measures taken from one arm (5a or 5b) and one leg (6a or 6b).

Evidence suggests that low oximetry levels are linked with a greater risk of delirium, [44] and while this technology could potentially improve delirium detection and monitoring as well as guide decisions about intervention, it must first be piloted before being introduced in a larger scale study. Oximetry values will be obtained for six different sites represented in Figure 1: low right frontal cerebral (1), high right frontal cerebral (2), low left frontal cerebral (3), high left frontal cerebral (4), one arm (5a or 5b), and one leg (6a or 6b). Electrodes will be placed on the higher and lower frontal cerebral areas to cover a larger zone. To obtain the _r_SO_2_ value for each of these six sites, the electrode is left in place for more than 20 seconds for stability of signal, after which the number appearing on the device is recorded. From these six _r_SO_2_ values, two mean scores are calculated: the total cerebral score being the mean score of all four cerebral sites, and the total peripheral score being the mean score of the two peripheral sites. Lower cerebral values and normal peripheral ones are indicative of poorer cerebral oxygenation not due to peripheral hypoperfusion. Given the novelty of cerebral _r_SO_2_ obtained using NIRS, we will describe the validity of this approach by contrasting the scores with three standard tools for the detection and assessment of delirium: the ICDSC, the Delirium Index (DI), and the Confusion Assessment Method (CAM-ICU). These tools will be used at Days 1, 2, and 3 following the onset of delirium.

The secondary objective to examine the preliminary effect of the intervention on patient and caregiver outcomes will be measured using different research tools, including questionnaires described in Table 1.

### Sample size

Patients with delirium at either of these post-surgical units will be randomized along with their caregivers to either usual care or experimental group (Table 1). A convenience sample of 30 patient-caregiver dyads will be recruited based on the specific published literature on pilot studies [45–48]. Because of the high involvement needed from caregivers in this intervention, the indicator chosen to determine the feasibility of the study was set as obtaining consent from a proportion of 75% of the caregivers approached to participate in the study. With such a proportion, 30 dyads is a sample large enough to estimate the proportion of caregivers that consent with a precision of ± 15.5% using a 95% confidence interval. In addition, the chosen sample size will enable us to highlight any trend or possible differences in patient and caregiver outcomes between the two study groups.

### Recruitment

#### Original protocol

Recruitment for this study poses several challenges because study participants include postoperative patients with delirium which makes them temporarily unable to give informed consent. In the original protocol, we planned a preoperative screening to include only patients with high risk of delirium. This was defined as the presence of at least three of the following risk factors: age ≥ 65 years, history of delirium, active smoker, consumption of at least three daily alcoholic beverages, or sensory impairment (sight, hearing). Postoperative delirium was defined as a score of ≥4 on the ICDSC [19] confirmed by a medical diagnosis of delirium recorded in the hospital chart. Since it is impossible to predict whether and when delirium will occur, two recruitment steps were planned.

Step 1 – All patients admitted to the pre-surgical unit at the study hospital will be assessed for study eligibility before their surgery by the nurse-mentor. The aim and requirement of the study protocol will be presented to eligible patients. Patients who agree will give the name of a family caregiver who will also be informed of the study. Both members of the dyad will be allowed to discuss their participation in study and sign the consent forms.

Step 2 – After surgery, if patients experience delirium, their family caregiver will be phoned by the nurse-mentor to re-confirm their initial consent, and the dyad will be included in the study. For dyads randomized to the intervention to be eligible, the caregiver must agree to participate within 24 hours of delirium onset in order to ensure a prompt start to the intervention.

Informed consent is obtained from each participant in this study.

#### Anticipated recruitment time

On average, 28 patients per week undergo CABG surgery or cardiac valve surgery with post-surgical recovery in the research hospital. A 40% incidence of delirium is estimated, which represents approximately 11 patients weekly. Our team’s past experience in the same research milieu with a study involving family caregivers in a highly similar context showed a recruitment rate of 30% [49]. Thus, a recruitment rate of one to two dyads weekly is expected and a total of 20 weeks is planned to reach a sample size of 30 dyads.

### Allocation

#### Sequence generation

The randomization scheme will be automatically generated by a coordinating center, and block of time allocation will be carried out using sealed, opaque envelopes.

#### Randomization

Given the setting of the research center’s ICU and post-ICU surgery wards, with one visitor room shared by both surgical units as the site of the nursing intervention, participant blinding to group assignment is not possible. This poses a risk of inter-group contamination. To reduce this risk, randomization of blocks of time will be used instead of individual randomization. Two-week blocks, followed by a washout period of one week will be assigned to either the control or intervention group. Thus, if a delirium episode is detected during a 2-week block allocated to control, this patient and their caregiver will be assigned to the control group and vice versa. Participants will not be informed of the present allocation of the 2-week block of time until after they have consented to the study. Delirium episodes detected during the 1-week wash out period will not be randomized or included in the study.

#### Blinding

While the nurse-mentor cannot be blinded to group assignment, ICU staff recording the usual care clinical data in the hospital chart (ICDSC, diagnosis of delirium) will be blinded, as well as the research assistant collecting outcome data. Finally, a numerical code will be assign to each group to allow blinding during data analysis.

### Data collection and analysis

#### Statistical methods

The planned analyses concerning all the primary objectives include descriptive statistics, which, along with 95% CI, will be used to portray the intervention and study design’s acceptability and feasibility criteria. Control and experimental group descriptions will be achieved using descriptive statistics reflected by n, mean, standard deviation, and median, minimum, and maximum for continuous variables and frequencies and percentages for categorical variables; 95% CIs will also be presented when appropriate. Finally, to evaluate the validity of cerebral _r_SO_2_, a Pearson or Spearman correlation will be used (depending on level of measurement) to correlate oximetry scores and scores obtained on the ICDSC, DI, and CAM-ICU at Days 1, 2, and 3 following the onset of delirium.

The planned analyses for the study’s secondary objective of preliminary efficacy will include the statistical methods detailed below. It should first be noted that this is a pilot study assessing the feasibility of a larger study and statistical analyses will be used to examine the preliminary effects of the intervention and determine any trend in the results as this study is not powered for statistical testing. For H1 relative to delirium severity, a repeated measure ANCOVA will be performed using the DI scores taken on Days 1, 2, and 3 following delirium onset. The initial scores on the ICDSC taken at delirium onset will be used as a covariate. Post-hoc tests will be performed if the omnibus test is significant for the group by time interaction. Such an interaction would imply that delirium severity is different between groups over the three days. Mixed models will be used so that a patient with missing data at Days 1, 2, and 3 following the onset of delirium will not be excluded from the analysis. The intent-to-treat principle will be respected; all the patients in the study will be included in the analysis according to their group of randomization. Because the pilot study will not be used to definitely assess the efficacy of the intervention (this would be the objective of the larger study), we plan to conduct a sensitivity analysis using the last observation carried forward.

For hypothesis H2 on adverse clinical complications following onset of delirium, a combined outcome measure will be used (mention in the hospital chart of sternal wound dehiscence, a fall, respiratory tract infection, or accidental removal of urinary catheter, drain, arterial line or endotracheal tube = yes/no) and groups will be compared using a χ^2^ test.

Student’s T-tests for independent groups will be used for hypothesis involving continuous variables H3 on length of ICU and total hospital stay, H4 on psycho-functional recovery 1 month after the onset of delirium, H5 on caregiver’s anxiety level, and H6 relative to caregiver’s sense of self-efficacy. The planned treatment for missing data for hypotheses H3 to H6 is assigning the value corresponding to the group mean.

Assumptions underlying the planned models will be checked and alternative methods may be used if more appropriate. A significance level of 0.05 will be used for all analyses.

### Ethical considerations

Institutional review board approval was obtained from the relevant hospital in January 2013 (Project number assigned by the Montreal Heart Institute Ethics committee: 2012–288, 1420). Measures to protect confidentiality of study participants were put in place; all information collected will be held securely, with an identification number assigned to each participant to protect confidentiality. Amendments to protocol will be communicated through additional information in the trial registration form on the website of http://www.controlled-trials.com. Finally, to our knowledge, the proposed intervention poses no risk to participants.

## Discussion

### Study limits

The pilot study’s aim is to assess the acceptability, feasibility, and preliminary efficacy of a nursing intervention enabling family caregivers to be involved in delirium management. The study also aims to provide evidence of the validity of cerebral _r_SO_2_ obtained by NIRS among patients with delirium. The preliminary effects will be evaluated for both patient outcomes (delirium length and severity, adverse clinical outcomes, length of ICU and hospital stays, psycho-functional recovery) as well as caregiver outcomes (anxiety and self-efficacy). This experimental nursing intervention was designed based on previous studies in the fields of delirium and dementia, as well as on clinical guidelines, and is grounded in three theoretical frameworks.

Throughout the design of this study protocol, an effort was made to reduce possible severe threats to validity identified by the Cochrane Collaboration [50]. These threats include issues related to: i) sequence generation and allocation concealment (selection bias); ii) potential threats to blinding of participants and outcomes; iii) between-group contamination (performance and detection bias); and finally iv) incomplete outcome data and selective outcome reporting (attrition and reporting bias).

In the present study, the sequence generation of the random group assignment will be carried out by experts from an independent clinical trial coordinating center (Montreal Health Innovations Coordinating Center). They will provide sealed opaque envelopes to be opened by the study nurse on the first Monday of the beginning of each 2-week block. In addition, a detailed flowchart of approached, randomized, and assigned participants will be included in the final report as well as a description of participants’ baseline characteristics.

Given the fact that blinding from group allocation is not possible for the patient, caregiver, and nurse-mentor, it is planned that all bedside nurses, medical team members, and research assistants be blinded. Nursing and medical staff will not be notified of week allocation, and the nurse-mentor will be present on the research wards during weeks allocated to both intervention and control groups. Therefore, delirium scores collected by the nurses will have been collected by professionals blinded to group assignment. The research assistant will also blinded to the study group when collecting data. The analyses will be conducted by an analyst blinded to group allocation insured by a codification of group allocation.

Another closely related potential bias is between-group contamination. This issue was addressed while planning the randomization scheme. The randomization of blocks of time followed by washout periods is designed to avoid this bias. In addition, pre- and post-visit encounters between the caregiver and the nurse-mentor will be held away from other families.

Finally, in an attempt to reduce potential issues with selective outcome reporting, all the planned feasibility and acceptability evaluation criteria as well as statistical analyses of outcomes have been described in the trial registration form (http://www.controlled-trials.com; #ISRCTN95736036).

However, despite this cautious approach in the study protocol, other threats to validity cannot be controlled. For instance, caregivers’ involvement in delirium management will result in an individualized dosage and quality of the intervention since all individuals have different resources to deal with this complex situation. To describe the intervention delivered, the caregiver’s presence and use of recommended behaviors will be closely monitored and reported; these data may also permit the exploration of the relationships between the specific types of intervention (e.g., type of caregiver behavior effectively performed, number of behaviors, number of encounters, duration of encounters) and the outcomes. These types of exploratory analysis serve to describe possible links between specific interventions and outcomes [16]. The use of only one study hospital poses another potential threat, this time to external validity. To minimize this bias, a thorough description of both the hospital setting and the type of patients will be provided. The fact that the intervention will be provided by one study nurse-mentor with an expertise in intensive care and delirium also limits the general application of the intervention by nurses with different expertise.

One last concern linked to the experimental intervention is its acceptability to caregivers and its feasibility in a context of delirium with a high variability of symptoms. A patient’s delirium is associated with high anxiety in caregivers [8–10] which could present a barrier to their involvement. Therefore, in evaluating the intervention’s acceptability, one main indicator was to obtain consent from at least 75% of the caregivers approached to participate in the study. The intervention’s feasibility will be evaluated based on the indicators presented in Table 4.

### Preliminary feasibility results

Within the first few months of the empirical phase we encountered challenges in the prediction and detection of delirium, as well as some ethical dilemmas. Because patients can show signs of delirium immediately upon waking from surgery, our original protocol mandated preoperative recruitment in order to ensure their informed consent before the surgery to participate in the study. In addition, in order to limit the number of patients approached before surgery our ethics committee suggested limiting screening to only those patients at high risk of developing delirium. Based on the available literature [4, 5, 51] and our clinical experience with delirium in the cardiac surgery population, it was hypothesized that patients with three or more of the risk factors for delirium (aged over 65, history of delirium, active smoking, consumption of at least three alcoholic beverages daily, or sensory impairment (sight, hearing)), would be more susceptible to developing post-surgical delirium. However, after a few weeks of recruitment it was evident that most delirium episodes occurred in patients with less than three risk factors (i.e., those from whom we did not have pre-operative consent), and did not necessarily occur in those with three or more. Therefore, we concluded that even though several risk factors for delirium have been identified [4, 5, 51], the surgery itself and age over 65 seem to be enough to trigger postoperative delirium in our patients. In light of this, the recruitment strategy protocol initially planned as a preoperative approach was amended with approval from the ethics committee to allow a postoperative approach, i.e., when delirium is observed after the surgery, in order to facilitate the inclusion of participants in the study.

The postoperative approach to recruitment implied specific ethical issues because patients with delirium lose their ability to give informed consent for the duration of their delirium. Given that the experimental intervention had little potential for deleterious effects, that a caregiver was involved, and that the patients would be able to consent after resolution of their delirium, the ethics committee allowed a new procedure for recruitment. This procedure included obtaining informed consent from the caregiver only during the delirium, followed by informed consent from the patient once the delirium resolved. The ethics committee’s decision was also based on a modified article of the Quebec Civil Code [52] which now allows the family to consent for the temporarily cognitively impaired parent in studies in which potential deleterious effects are minimal. This is parallel to the consent for care that can be provided by family members in clinical care settings when the ill person is unable to consent due to cognitive impairment. Therefore, in the present study, we obtain informed consent from caregivers when patients themselves are unable to consent. The patient has to consent for the study after emerging from the delirium before the time or outcome assessment at 1 month; the data collected in patients who cannot consent within that time frame are excluded from the study, as well as those for caregivers. This approach to recruiting patients is proving more effective than the initial approach.

In addition to this first challenge, our team experienced difficulty with delirium detection. In the research milieu, a delirium screening tool, the ICDSC, is administered three times a day as part of usual care for all post-surgical patients in the ICU, and as needed on the surgical ward. It was expected that delirious patients would be identified by a score of four or higher on the ICDSC, as recommended [19]. However, we observed that a number of patients with hypoactive delirium do not score four or more on the ICDSC. Hypoactive delirium is recognized by the scientific community as being highly underdiagnosed or misdiagnosed for depression because of its less behaviorally-disturbing nature [53, 54]. Until now, the most effective way to identify patients with hypoactive delirium has been to speak to all patients with a score of at least one on the ICDSC scale, and focus on identifying more subtle symptoms such as inattention or mild hallucinations.

It has been established that delirium results in negative consequences for patients, leaving them with psycho-functional sequelae [5, 6, 10], as well as for families who report high anxiety [10] and for the health care system [55]. The search for new strategies for delirium prevention, management, and monitoring, eventually leading to better outcomes for all areas affected by this serious syndrome, is pressing. The proposed pilot study protocol will enable us to build a larger study with a sufficient number of participants to ensure adequate statistical power that can potentially add to the knowledge on delirium management and monitoring. Another potential important contribution of this pilot study is in the validation of cerebral oximetry with NIRS. This promising physiologic measure of cerebral perfusion could fill a gap in providing an objective indicator for early detection and continuous monitoring of delirium. Finally, this study is proposed to assess the feasibility and acceptability of the study design and intervention which could potentially add to the literature on pilot studies because it is based on the recent literature on this research strategy [45–48].

## Trial status

The trial is currently recruiting participants.

## Authors’ original submitted files for images

Below are the links to the authors’ original submitted files for images. Authors’ original file for figure 1 Authors’ original file for figure 2

## Abbreviations

CABG: Coronary artery bypass graft surgery
CAM-ICU: Confusion assessment method-intensive care unit
CI: Confidence intervals
DI: Delirium index
DSM-V: Diagnostic and statistical manual of mental disorders fifth edition
ICDSC: Intensive care delirium screening checklist
ICU: Intensive care unit
NIRS: Near infrared spectroscopy
_r_SO_2_: Regional oximetry.
