# Supplementary figures and images for: Evaluation of a nurse mentoring intervention to family caregivers in the management of delirium after cardiac surgery (MENTOR_D): a study protocol for a randomized controlled pilot trial
Source: Trials. 2014 Jul 30;15:306. doi: 10.1186/1745-6215-15-306 (PMC4133622; doi:10.1186/1745-6215-15-306)

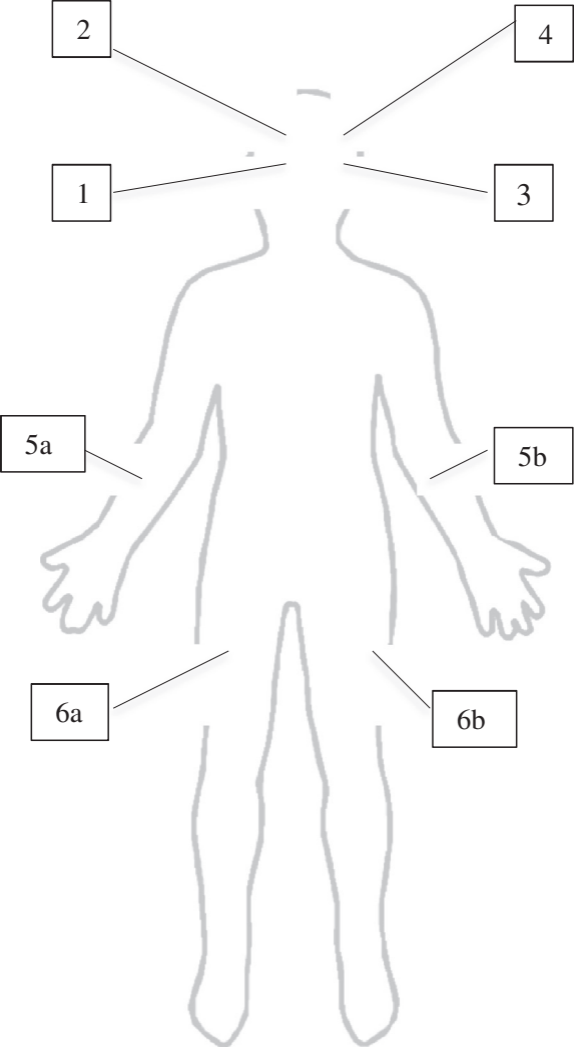

Supplement: Supplementary file 1 — Authors’ original file for figure 1 [file 13063_2014_2190_MOESM1_ESM.pdf]

**Figure 2 - Oximetry measures sites**

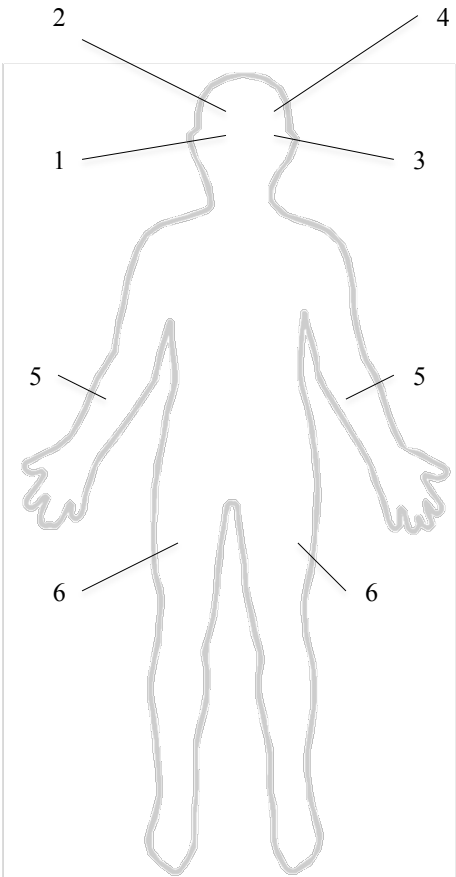

Supplement: Supplementary file 2 — Authors’ original file for figure 2 [file 13063_2014_2190_MOESM2_ESM.pdf]
